# Inflammation in Disease: Mechanism and Therapies

**DOI:** 10.1155/2013/829209

**Published:** 2013-06-27

**Authors:** Gustavo Duarte Pimentel, Marília Seelaender, Fábio Santos Lira

**Affiliations:** ^1^Department of Internal Medicine, State University of Campinas, 13083-970 Campinas, SP, Brazil; ^2^Cancer Metabolism Research Group, Institute of Biomedical Sciences, University of São Paulo, 05508-900 São Paulo, SP, Brazil; ^3^Laboratory of Exercise Biochemistry and Physiology, Health Sciences Unit, University of Southern Santa Catarina, 88806-000 Criciúma, SC, Brazil

Inflammation in the face of harming stimuli protects the organism; as a result, it is an essential process for survival, in which both innate and adaptive immunity are involved. This process must be tightly controlled and terminated in order to warrant the reestablishment of body homeostasis. Therefore, activation of resident inflammatory cells and the recruitment and modulation of migrating inflammatory cells must be ceased. When failure in neutralising acute inflammation occurs, there is augmented risk for the development of chronic inflammation, leading to several metabolic consequences. In the present special issue, original research studies as well as review articles address the inflammatory process as a key contributor to the development of disease. In addition, pharmacological and nonpharmacological therapies are examined and the molecular and physiological mechanisms of the treatments are discussed.

Among the 24 accepted articles, 5 focus on nutritional therapy and inflammation (D. Estadella et al.; L. Ma et al.; M. J. Kim et al.; S. Scolleta et al.; C. Cunha et al.). The selected articles discuss the effects of the consumption of saturated and trans fatty acids upon tissue lipotoxicity, the influence of resveratrol on lung injury, the beneficial effect of genistein on renal damage, the role of vitamin D in the resolution of inflammation, and finally the capacity of green tea extract to counteract the consequences of obesity.

The ability of exercise to modulate chronic inflammation is the focus of two articles, the first of which discusses the adaptive response of skeletal muscle to inflammation as induced by eccentric overload resistance training (B. N. Ide et al.), while the other considers exercise effects upon spinal cord injury-associated low grade inflammation (E. S. Alves et al.).

Pulmonary diseases and inflammation are the subject debated in 8 from the total of 24 articles selected for this issue, with special emphasis on the possible triggers of inflammation onset in the scenario (Y. Yang et al.; G. Karakiulakis et al.; M. S. Duan et al.; Geve et al.; I. T. Lee et al.; P. Seidel et al.; Y. Shimizu et al.; A. D. Yalcin et al.). In fact, asthma, emphysema, and COPD are extensively discussed in this issue, and studies concerning the effects of treatment with dimethyl fumarate, anti-IgE; activation of muscarinic receptors of immune cells; and proinflammatory signalling are reviewed. 

Sleep disorders are also contemplated in articles (V. A. Lemos et al.; D. Rosa et al.; Y. Jin et al.) that demonstrate that sleep apnea contributes to the increased inflammatory cytokine content, a response to hypoxia induced by high altitude.

Finally, 3 studies investigate the role of PPAR, 41BB/4 1 BBL, and mitochondrial dysfunction in metabolic disease (T. H. Tu et al.; F. Monsalve et al.; A. Hernández-Aguilera et al.). In addition, inflammation is discussed in the presence of periodontal disease and endotoxaemic shock (J. D. Corrêa et al.; A. Sampaio et al.). 

Collectively, this issue aims at providing insight on the role of acute and chronic inflammation in different diseases, as well as at presenting recently proposed treatment strategies.

*Gustavo Duarte Pimentel* *Gustavo Duarte Pimentel* *Marília Seelaender* *Marília Seelaender* *Fábio Santos Lira* *Fábio Santos Lira*

